# Our Officinal Cathartic Pills

**Published:** 1871-03

**Authors:** E. Ingals

**Affiliations:** Chicago


*Our Officinal Cathartic Pills.* By E. INGALS, M.D., of Chicago.

The cathartic pills made officinal by our pharmacopœia, appear to me to lack the properties which the necessities of the profession require in such preparations, namely, efficiency and gentleness of action, the compass small, the ingredients inexpensive.

The following are some of the points that may be urged against them. They all require so large a number to produce cathartic action, as to constitute with most patients a serious objection to their use, and the poor quality of the drugs often employed in compounding them serves but to increase the weight of this objection. In a number of them aloes is the chief ingredient, and cathartics are often required when the conditions of the large intestines and pelvic viscera contra-indicate the use of this article, at least in such doses as are usually prescribed. Rhubarb, and the compound extract of colocynth, are expensive, and therefore often adulterated, a choice article of either costing twice as much as that usually kept in the shops.

The suggestion of a cheap remedy is a matter of more than pecuniary significance, for the maxim of Pliny, "the poorer the purer," applies with equal force to other articles than stimulating beverages. Against the compound cathartic pills lies the more serious objection that each pill contains one grain of calomel. These, I fear, are often prescribed because they are officinal, and stand ready on the shelf of the pharmaceutist, when the physician would not think of writing for calomel in a prescription for his patient—but the practice, first adopted from convenience, soon becomes a habit.

Mercurials should never be prescribed unless the physician has in his mind a clear reason for supposing they will accomplish some desired purpose better than any other article; but to administer them as cathartics are most frequently used—simply to unload the bowels, to deplete the system, or obviate constipation—is altogether unwarrantable, and I doubt not this practice sometimes results in permanent injury to the constitutions of the sick.

Seeking to supply this desideratum, I have made trial of a number of substances, united in different proportions, but the following formula has proved to me the most satisfactory:

| | | |
|---|---|---|
| ℞. Aqueous Extract of Aloes, Podophylin, Gamboge, | - - - | āā gr. xxx. |
| Oil of Caraway, - - - - - - - | | m. vj. |
| M. ft. pil. No. lx. | | |

I have had the pills made up and sugar-coated in a very satisfactory manner by J. W. Ehrman, a pharmaceutist of this city. The pill is small, weighing only three grains after being sugar-coated. One taken at bed-time will usually cause an evacuation from the bowels in the morning, and without occasioning either pain or nausea; but should one fail, or be thought insufficient, another may then be taken. I have rarely found it necessary to administer more than two, and for some patients who are peculiarly susceptible to the action of cathartics, the half of one is sufficient. The aqueous extract of aloes seems to contain the more cathartic principles of the drug, while it is the least irritant to the bowels, and the amount administered being so small, it does not appear to act injuriously, even in cases in which the effect of the ordinary dose of aloes is unpleasant.

Could some formula similar to the one I have indicated, be placed in the next revised edition of the pharmacopœia, I believe it would in a great degree supplant the use of the compound cathartic pill, and by so doing, prove a benefit both to the public and profession.

---

### *Belladonna in Cholera Morbus.*

Dr. H. E. Whitehead having had occasion to treat several cases of severe attacks of cholera morbus, and having failed to relieve the first case with opium, ordered for the patient, pills containing one-third to one-half a grain of the extract of belladonna; one to be taken every four hours. The first pill relieved the sense of constriction or knotty pain in the region of the umbilicus, and after the third pill the bowels were freely moved, although several purgative draughts had been taken previously without effect. After having taken two grains of the extract the patient rapidly recovered, with no constipation, headache, or unpleasant symptoms. The result in three other cases, with the belladonna treatment, was equally successful.—*Pacific Medical and Surgical Journal.*

